# Metastatic Retroperitoneal Rhabdomyosarcoma in a Child: A Case Report Highlighting the Role of Imaging in Diagnosis, Staging, and Follow-Up

**DOI:** 10.7759/cureus.115248

**Published:** 2026-08-26

**Authors:** Samia Obilat, Lina Lasri, Nazik Allali, Latifa Chat, Siham Elhaddad

**Affiliations:** 1 Department of Radiology, Children's Hospital of Rabat, Mohammed V University, Rabat, MAR

**Keywords:** computed tomography, image-guided biopsy, pediatric oncology, retroperitoneal mass, rhabdomyosarcoma (rms), sarcoma staging

## Abstract

Retroperitoneal masses in children encompass a heterogeneous group of benign and malignant tumors with a largely nonspecific clinical presentation. Rhabdomyosarcoma (RMS) is a soft tissue sarcoma that uncommonly arises primarily in the retroperitoneum, a site associated with a comparatively unfavorable prognosis related to delayed diagnosis, large tumor volume at presentation, and frequent invasion of adjacent vascular and digestive structures.

We report the case of a five-year-eight-month-old boy who presented with a one-month history of progressive abdominal distension and left lower limb edema secondary to iliac vein compression. Cross-sectional imaging revealed a large heterogeneous retroperitoneal mass with pulmonary and osseous metastases at diagnosis. The initial percutaneous biopsy was morphologically consistent with embryonal RMS, while a repeat biopsy was non-representative. The patient received induction chemotherapy with a 96.2% estimated volumetric response, followed by conservative local surgical management. Approximately one year after diagnosis, he experienced severe clinical deterioration and subsequently died.

This case illustrates the diagnostic challenges of retroperitoneal RMS in children, the pitfalls of percutaneous biopsy in large heterogeneous tumors, and the central role of imaging at every stage of diagnosis, treatment response assessment, and follow-up, despite the unfavorable prognosis of this rare tumor site.

## Introduction

Retroperitoneal masses in children are a heterogeneous group of tumors that most often present nonspecifically, with progressive abdominal distension, an incidentally discovered palpable mass, or abdominal pain [1]. Rhabdomyosarcoma (RMS), the most common soft tissue sarcoma of childhood, comprises over 50% of pediatric soft tissue sarcomas [2,3] and accounts for 5%-8% of childhood cancers [1], with a marked predilection for the head and neck and the genitourinary tract; primary retroperitoneal involvement is exceptional, representing only 6%-8% of RMS sites [4]. Despite this rarity, retroperitoneal RMS carries one of the poorest prognoses among primary RMS sites, related to delayed diagnosis, large tumor volume at presentation, and frequent involvement of adjacent vascular and digestive structures that may compromise complete local control [5,6].

Cross-sectional imaging plays a central role in characterizing the mass, defining its anatomical relationships, narrowing the differential diagnosis, and staging [1,7]. However, the imaging appearance of retroperitoneal RMS may overlap with that of more common pediatric retroperitoneal malignancies, particularly when no definite organ of origin is identified. In addition, intratumoral heterogeneity in soft-tissue sarcomas may complicate percutaneous tissue sampling and result in non-representative specimens [8].

We report a case of metastatic embryonal retroperitoneal RMS in a child to illustrate the interaction between radiological phenotype, differential diagnosis, biopsy sampling, metastatic staging, and longitudinal imaging assessment throughout treatment and follow-up.

## Case presentation

Clinical presentation

A five-year-eight-month-old boy with an unremarkable medical history presented with a one-month history of progressive abdominal distension without fever, prompting referral to our institution for further evaluation.

Physical examination showed an afebrile, hemodynamically stable child with a firm abdominopelvic mass measuring approximately 10 cm, collateral abdominal venous circulation, and left lower limb edema. Small bilateral inguinal lymph nodes were also noted; on initial CT, the largest measured 9 mm in short-axis diameter on the left, without suspicious imaging features, and was considered reactive. Moderate-to-severe malnutrition with mild growth delay required transient enteral nutritional support.

Laboratory tests showed moderate anemia, an elevated C-reactive protein level, mild hyperuricemia, and markedly elevated lactate dehydrogenase (LDH) in the setting of a large tumor burden, with preserved renal and hepatic function (Table 1). Bone marrow aspiration showed no evidence of malignant infiltration.

**Table 1:** Laboratory results at admission AST, aspartate aminotransferase; SGOT, serum glutamic-oxaloacetic transaminase; ALT, alanine aminotransferase; SGPT, serum glutamic-pyruvic transaminase

Parameter	Patient's value	Unit	Reference range
Hemoglobin	9.8	g/dL	11.5-14.5
C-reactive protein	52.7	mg/L	<5
Uric acid	74.5	mg/L	35-72
Lactate dehydrogenase	650	U/L	125-220
Creatinine	5.9	mg/L	7.2-12.5
AST (SGOT)	26	U/L	5-34
ALT (SGPT)	5	U/L	0-55

Imaging findings

**Figure 1:**
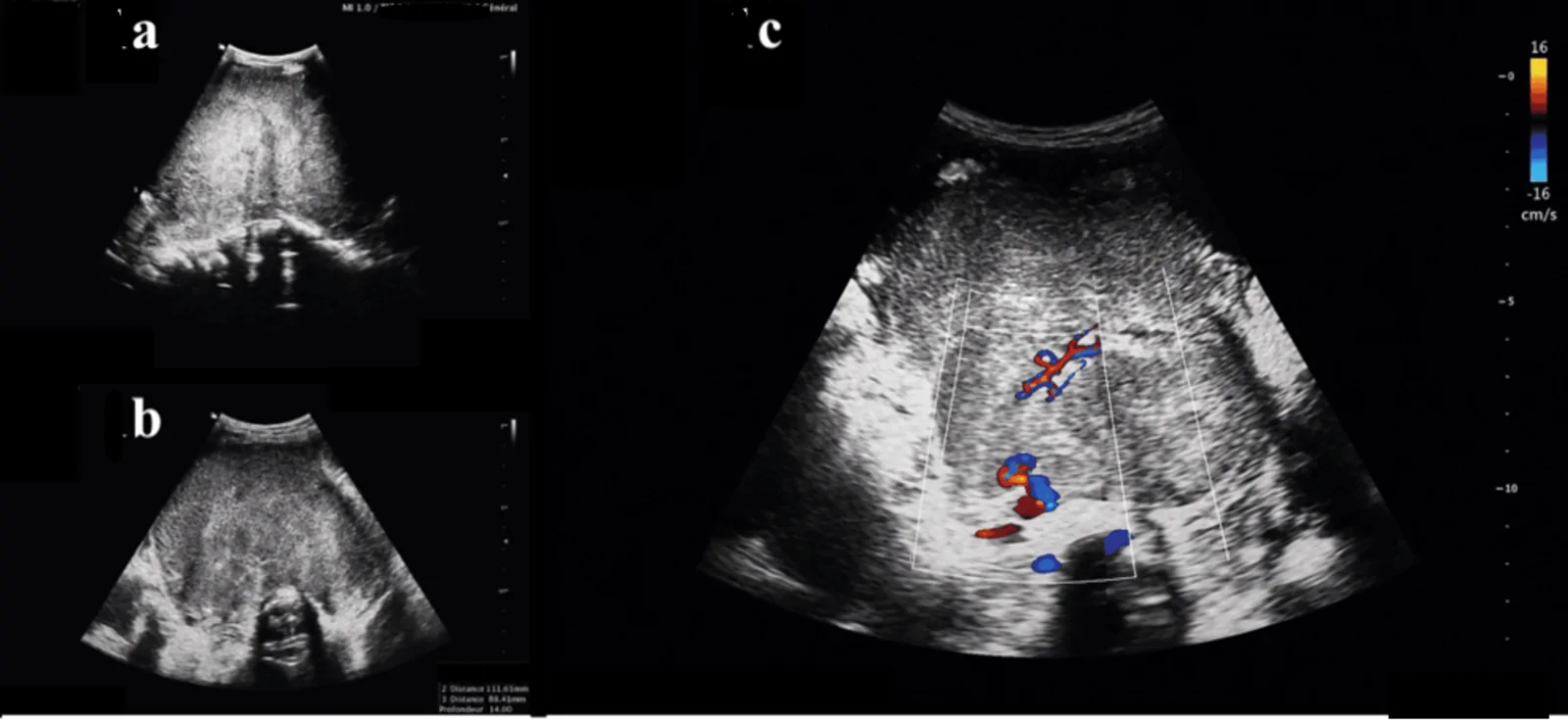
Initial abdominal ultrasound. (a and b) Grayscale image showing a large, well-defined, heterogeneous prevertebral retroperitoneal mass. (c) Color Doppler image showing sparse internal vascularity.

Abdominal ultrasound showed a well-defined, heterogeneous, poorly vascularized prevertebral retroperitoneal mass measuring approximately 111 × 88 × 152 mm, exerting mass effect on the aorta and inferior vena cava (Figure 1), with bilateral uretero-hydronephrosis and moderate peritoneal effusion.

Contrast-enhanced thoraco-abdomino-pelvic CT confirmed a large prevertebral retroperitoneal mass with no definite organ of origin, both kidneys and adrenal glands being clearly distinct from the lesion. On retrospective review, the mass measured 114 × 121 × 150 mm. It was predominantly solid and markedly heterogeneous, without calcification, containing nonenhancing low-attenuation areas, suggestive of tumor necrosis. It extended from approximately the L1 level to the pelvis, displacing the bowel loops and bladder anteriorly and to the right. Inferiorly, it was in close contact with the rectum, with focal loss of the intervening fat plane.

The mass demonstrated extensive vascular and muscular involvement. It contacted the infrarenal abdominal aorta over more than 180° and displaced the aorta and inferior vena cava to the right. It encased the left common, internal, and external iliac arteries and was in broad contact with the corresponding right iliac vessels. The left iliac venous axis was also involved, with extrinsic compression of the left external iliac vein without thrombosis, accounting for the left lower limb edema. The mass was focally inseparable from the left psoas and ipsilateral iliacus muscles, consistent with direct muscular invasion. Bilateral uretero-hydronephrosis was present secondary to pelvic mass effect.

**Figure 2:**
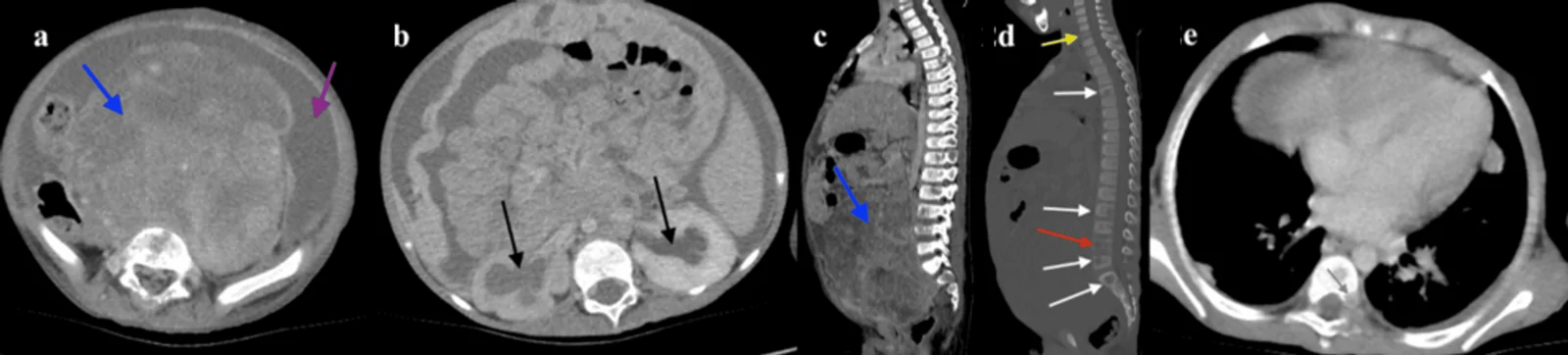
Initial contrast-enhanced abdominopelvic CT. (a) Axial image at the portal venous phase showing the large heterogeneous retroperitoneal mass (blue arrow) with a peritoneal effusion (purple arrow). (b) Axial image showing mass effect on adjacent structures with bilateral hydronephrosis (black arrows). (c) Sagittal reformation showing the cranio-caudal extent of the mass (blue arrow). (d) Sagittal bone-window CT image demonstrating multilevel lytic metastatic involvement of the lumbosacral spine (white arrows), with a pathologic compression fracture of L4 (red arrow). A C6 compression deformity is also present (yellow arrow), without a definite associated focal osseous lesion to establish its metastatic nature. (e) Axial bone-window CT image at the T5 level showing a lytic soft-tissue metastasis centered on the left pedicle, extending to the ipsilateral transverse process and vertebral lamina and into the spinal canal (brown arrow).

CT also demonstrated extensive osseous metastatic disease, with lytic soft-tissue lesions involving the L2-S1 vertebrae and an associated pathologic compression fracture of L4. Additional lytic soft-tissue lesions centered on the pedicles of T5 and T12 extended to the ipsilateral transverse processes and vertebral laminae, with extension into the spinal canal. Further osseous lesions involved the iliac wings and the femoral heads and necks. A C6 compression deformity was also present at baseline, although no associated focal osseous lesion was identified to confidently characterize it as metastatic (Figure 2).

**Figure 3:**
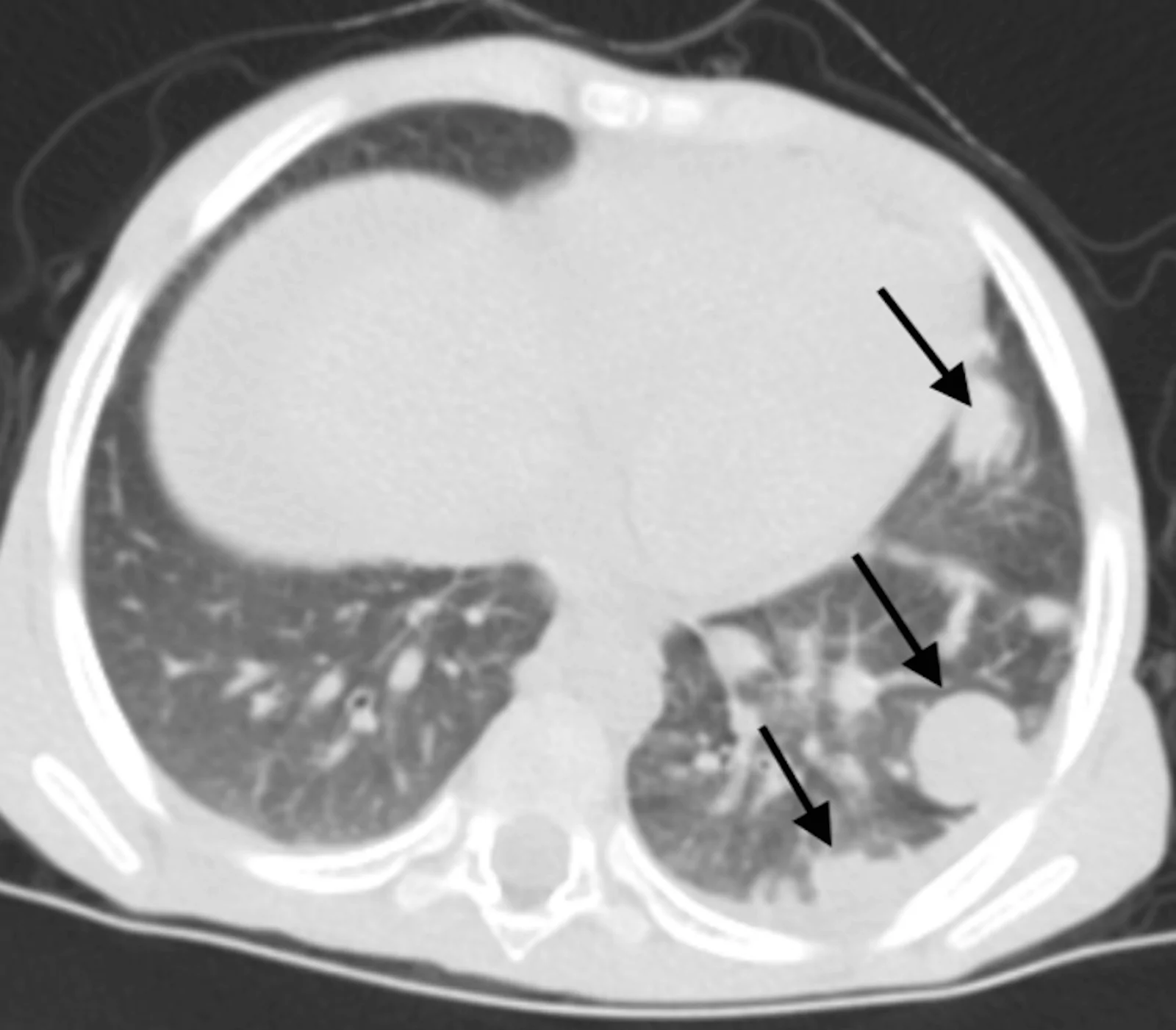
Initial chest CT, lung window. Multiple bilateral subpleural pulmonary nodules (black arrows) compatible with metastatic disease.

Chest CT showed multiple bilateral, predominantly subpleural, solid pulmonary nodules, the largest measuring approximately 16 mm, associated with nodular pleural thickening and a thin left pleural effusion, consistent with thoracic metastatic disease (Figure 3). No mediastinal or axillary lymphadenopathy was identified.

Histopathological findings

**Figure 4:**
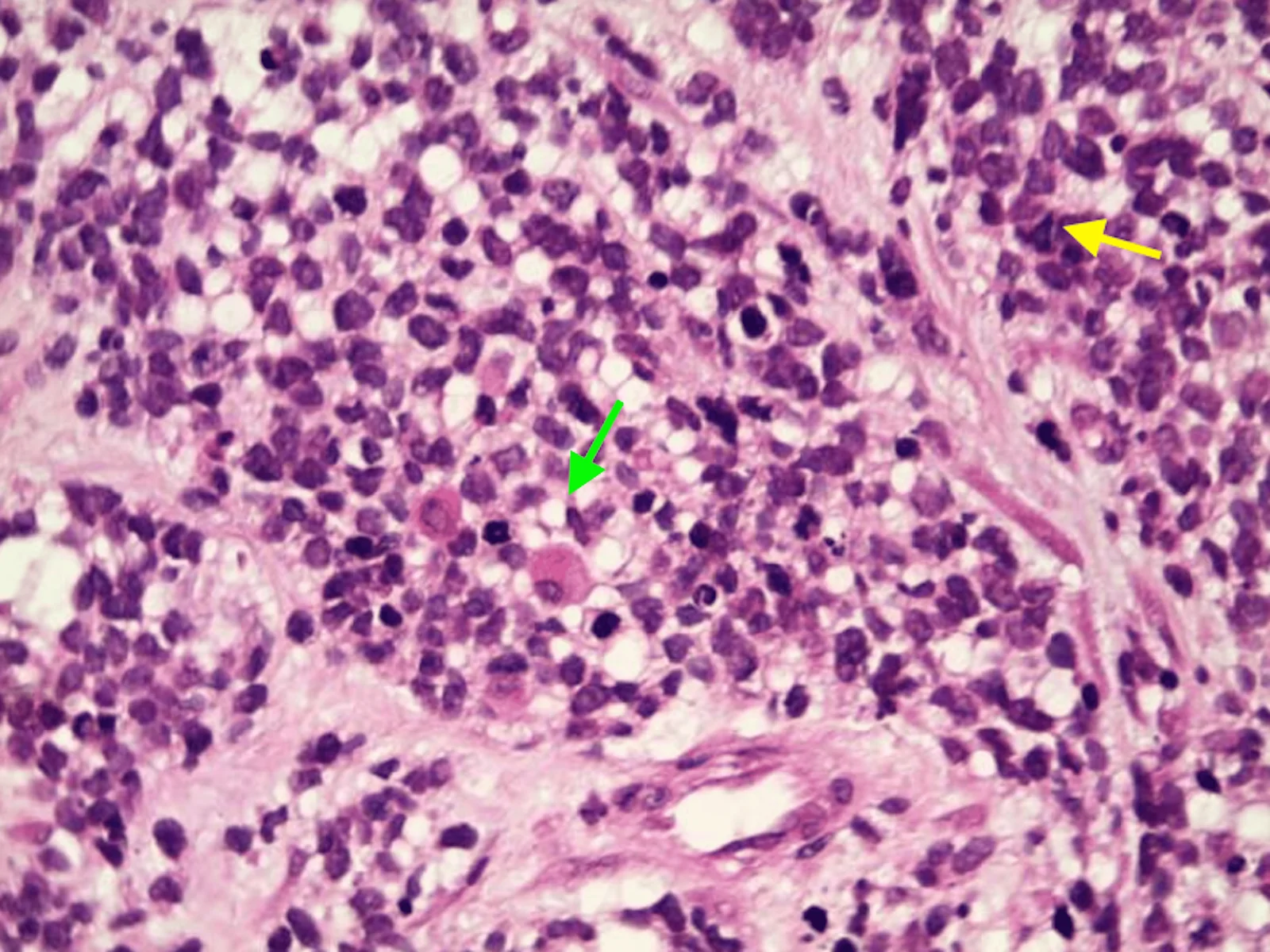
Histopathological findings of the initial percutaneous biopsy. Hematoxylin and eosin staining (original magnification ×100) showing a proliferation of round-to-spindle cells with hyperchromatic nuclei arranged in sheets within a myxoid stroma with delicate fibrovascular septa (yellow arrow), and a focal rhabdomyoblast with abundant eosinophilic cytoplasm (green arrow), consistent with embryonal rhabdomyosarcoma.

Ultrasound-guided core needle biopsy of the retroperitoneal mass was performed. Three core specimens measuring 1-1.2 cm were obtained and were entirely tumor-containing. Histological examination showed a proliferation of small round to spindle-shaped cells with hyperchromatic nuclei and scant cytoplasm, arranged in sheets on a myxoid background. Foci of rhabdomyoblastic differentiation were identified, characterized by eccentrically located nuclei and abundant eosinophilic cytoplasm with cross-striations (Figure 4).

These morphological findings were consistent with embryonal RMS. Immunohistochemistry was attempted but was technically inconclusive because of tissue section detachment and excessive specimen thickness. A repeat biopsy was therefore recommended to obtain immunohistochemical confirmation.

A second ultrasound-guided core needle biopsy yielded three whitish-translucent fragments measuring 0.8-1.2 cm. Histological examination demonstrated fibro-hyalinized tissue containing sparse spindle cells with regular nuclei and no identifiable mitotic activity. Immunohistochemical staining for desmin and myogenin was negative, and no viable tumor was identified in the submitted fragments. In the context of the diagnostic morphology of the initial biopsy and the marked radiological heterogeneity of the mass, the second biopsy was considered non-representative rather than evidence against the initial diagnosis.

Staging, treatment, and follow-up

**Figure 5:**
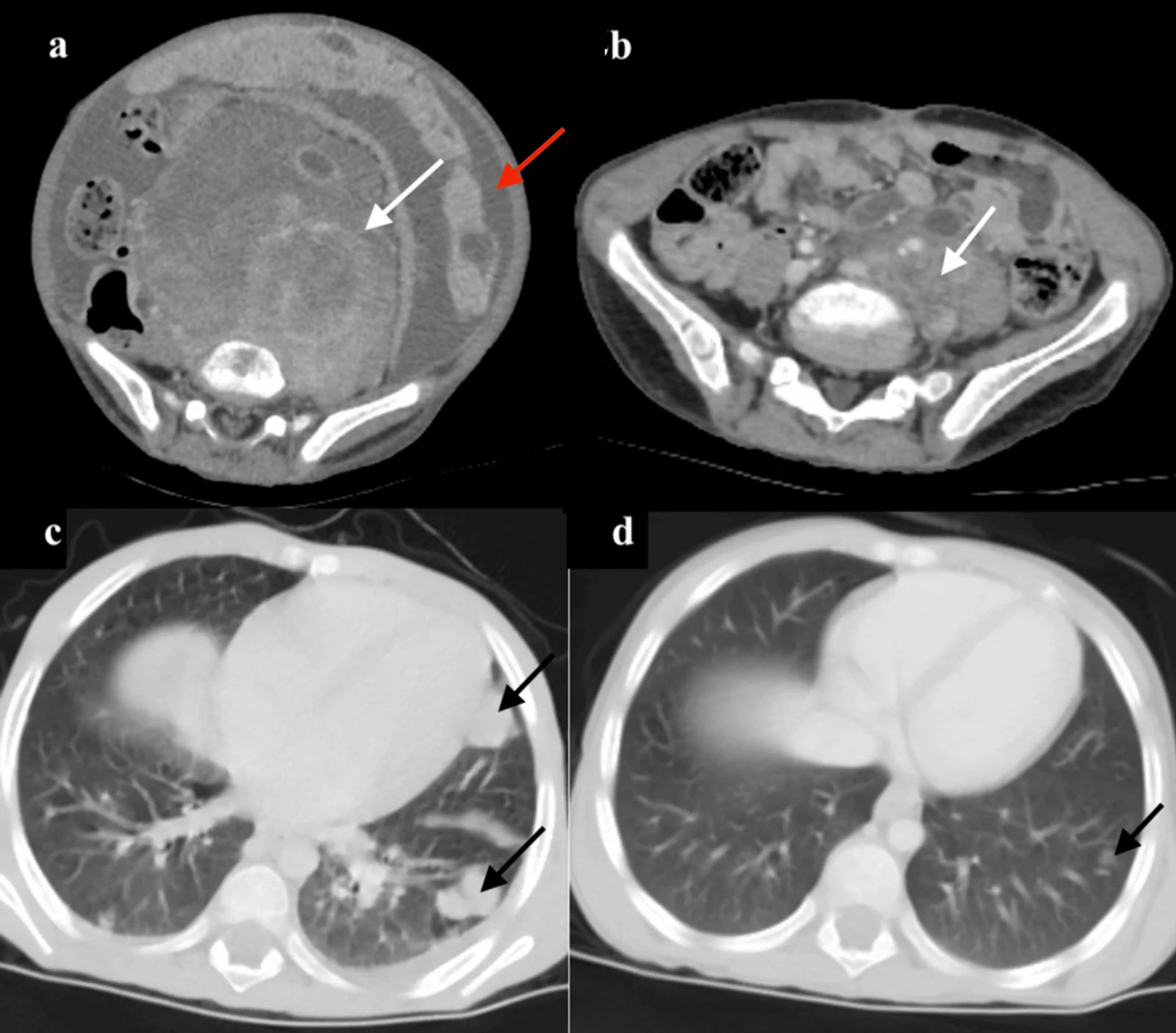
Comparative CT before (a, c) and after (b, d) induction chemotherapy. (a, b) Abdominopelvic CT showing marked regression of the retroperitoneal mass (white arrow) and resolution of the peritoneal effusion (red arrow). (c, d) Chest CT showing near-complete resolution of the pulmonary nodules (black arrows).

The final diagnosis was metastatic embryonal retroperitoneal RMS with pulmonary and osseous metastases. Pretreatment staging was modified TNM stage 4 and Intergroup Rhabdomyosarcoma Study (IRS) clinical group IV. After four cycles of induction chemotherapy with alternating IVADO (ifosfamide, vincristine, actinomycin D, and doxorubicin) and IVA (ifosfamide, vincristine, and actinomycin D) regimens, CT demonstrated a marked treatment response. Using an ellipsoid volume approximation based on three orthogonal tumor measurements, the estimated tumor volume decreased from approximately 1,083 cm³ at baseline (114 × 121 × 150 mm) to 41.5 cm³ after induction chemotherapy (41 × 23 × 84 mm), corresponding to a 96.2% volumetric reduction. Thoracic metastatic disease also markedly regressed, with disappearance of the pleural nodules and persistence of only a small left posterobasal pulmonary nodule (Figure 5).

Following the marked response to induction chemotherapy, local surgical management was undertaken. A small fibrous residual tissue adherent to the left ureterovesical junction was identified intraoperatively and managed conservatively with ureterolysis and metallic clip marking of the tumor bed. No intraoperative biopsy of this residual tissue was performed; therefore, its viability could not be histologically assessed. Locoregional radiotherapy was subsequently considered but could not be delivered because the irradiation volume required to encompass the initially extensive disease was considered too large by the radiation oncology team. Given the persistent metastatic disease and the limitations of locoregional treatment, metronomic cyclophosphamide monotherapy was initiated as maintenance therapy.

**Figure 6:**
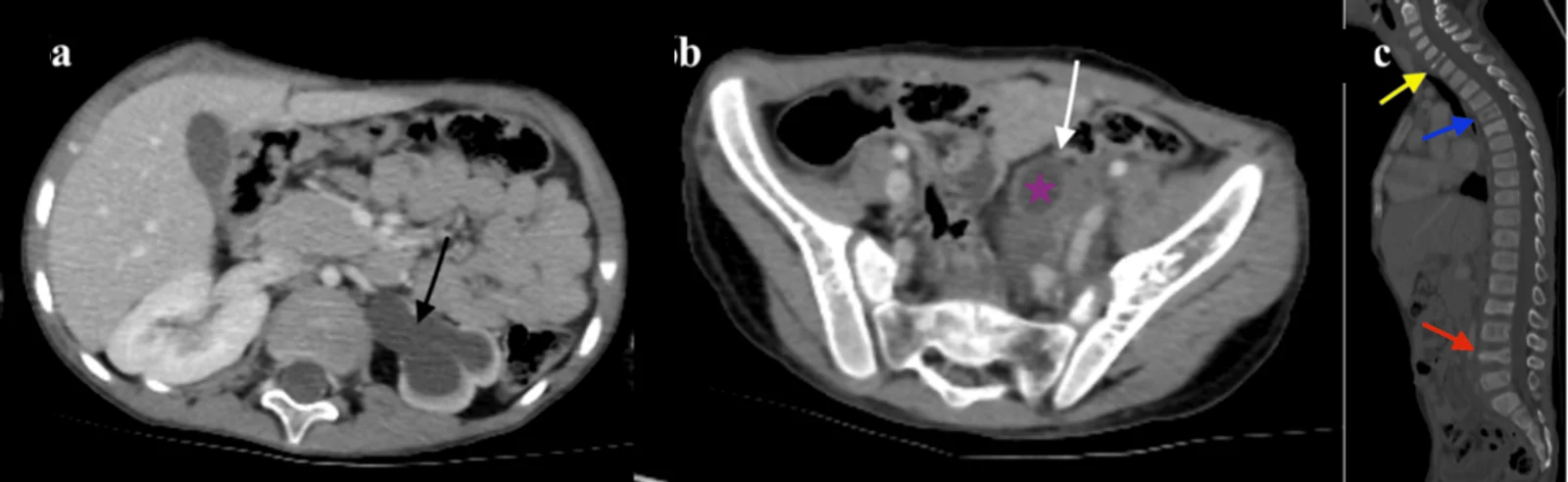
Imaging findings during follow-up. (a) Axial contrast-enhanced CT image showing persistent left ureterohydronephrosis (black arrow). (b) Axial contrast-enhanced CT image showing the residual retroperitoneal tumor tissue (white arrow) adjacent to the dilated left ureter (purple asterisk). (c) Sagittal bone-window reformation of the entire spine showing progression of the L4 compression fracture (red arrow) and a new dorsal lytic lesion (blue arrow), with an unchanged cervical compression fracture (yellow arrow), compared with the initial examination (Figure 2d).

On follow-up imaging, right ureterohydronephrosis regressed in parallel with the primary mass, whereas left ureterohydronephrosis persisted because of residual periureteral tissue encasing the left pelvic ureter, eventually resulting in chronic left renal atrophy with asymmetric excretory function. Serial imaging showed no significant increase in the residual retroperitoneal tissue. Follow-up bone-window CT demonstrated progression of the pathologic L4 compression fracture and additional multilevel vertebral compression deformities, without posterior vertebral wall retropulsion and new lytic lesions (Figure 6). 

Pulmonary nodules progressively resolved on follow-up chest CT.

**Figure 7:**
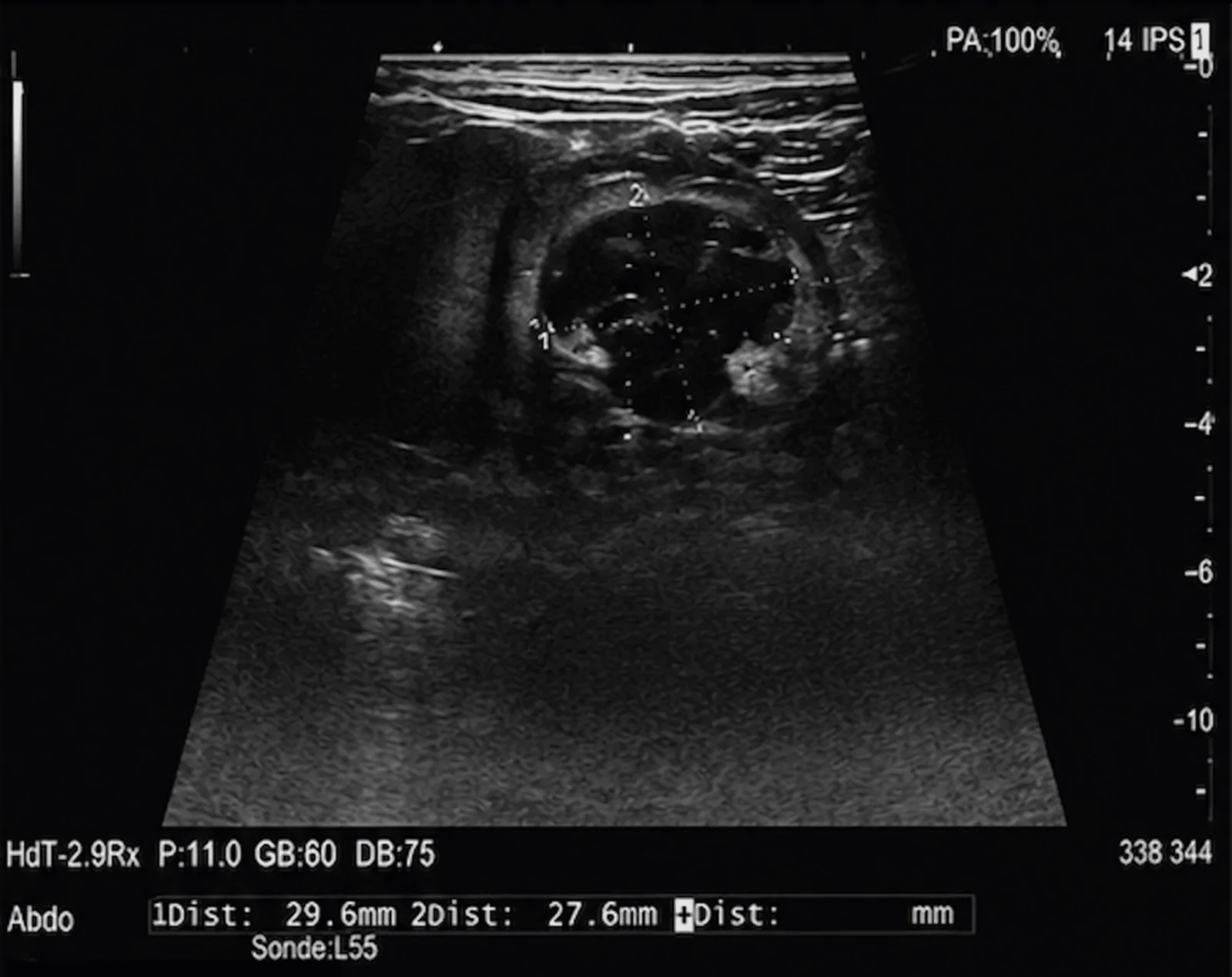
Follow-up abdominal ultrasound. Small necrotic-appearing residual retroperitoneal mass.

On serial ultrasound, the residual retroperitoneal tissue remained stable in size, with a persistently necrotic appearance (Figure 7).

Approximately one year after diagnosis, the child was admitted for abdominal pain, anorexia, and marked deterioration of general condition, with pallor, hepatomegaly, and a tender distended abdomen. Despite symptomatic management, his condition rapidly progressed over a few hours to a shock state consistent with terminal terminal shock state. The family was informed of the prognosis, and the child died at home a few hours after discharge at their request. No further imaging was available to determine whether this terminal clinical deterioration was associated with disease progression.

## Discussion

Our patient's presentation, progressive abdominal distension over about one month, matches the dominant mode of discovery of pediatric retroperitoneal sarcomas, reported as abdominal mass or distention in up to 76% of cases, ahead of abdominal pain (62%) [6]. Left lower limb edema from external iliac vein compression, by contrast, was a comparatively atypical finding: vascular involvement of the great vessels (iliac vein, inferior vena cava, aorta) has been reported in about one-fifth of pediatric retroperitoneal sarcomas, but compressive limb edema is rarely singled out among presenting signs [2,6].

A large retroperitoneal mass in a child raises a broad differential diagnosis, dominated by neuroblastoma, the most common malignant retroperitoneal tumor at this age, but also including nephroblastoma, germ cell tumors, and lymphoma [1]. Neuroblastoma typically arises from the adrenal gland or paraspinal sympathetic chain, is calcified in 80%-90% of cases, and tends to encase rather than invade adjacent vessels [1]. Nephroblastoma, by contrast, typically displaces rather than encases adjacent vessels, with calcification present in only about 10% of cases [9]. Germ cell tumors typically contain fat, fluid, or amorphous to spiculated calcification, while lymphoma tends to be homogeneous, presenting as a single or bilateral mass along the great vessels with comparatively little necrosis or calcification [1]. RMS, by contrast, typically presents as a bulky, non-calcified, heterogeneous mass, often with areas of necrosis and aggressive involvement of adjacent structures, including vessels [1]. As in most pediatric retroperitoneal masses, neuroblastoma was initially favored in our patient given its higher prevalence at this age. However, retrospective review demonstrated no definite organ of origin, with both adrenal glands and kidneys clearly separate from the mass. The absence of calcification, marked tumor heterogeneity, encasement of the iliac vessels, and direct invasion of the left psoas and iliacus muscles were more suggestive of an aggressive soft-tissue sarcoma such as RMS. Nevertheless, substantial imaging overlap remains between these entities, making histological confirmation mandatory.

A second diagnostic difficulty arose from the discordant biopsy findings. The initial biopsy was morphologically consistent with embryonal RMS, although immunohistochemistry was technically inconclusive. The repeat biopsy contained only fibro-hyalinized tissue without identifiable viable tumor. In the context of the marked radiological heterogeneity of the mass, this second specimen was considered non-representative rather than contradictory to the initial morphological diagnosis. This illustrates a recognized limitation of core needle biopsy in heterogeneous soft-tissue sarcomas [8] and highlights the importance of radiological-pathological correlation when biopsy findings are discordant. Retaining the diagnosis on the first sample avoided a potentially harmful delay in treatment.

Staging placed our patient in modified TNM stage 4 and IRS clinical group IV, defined by the presence of distant metastatic disease at onset [10], corresponding to a high-risk group associated with a three-year failure-free survival below 30% [2]. However, applying the Oberlin metastatic prognostic score, based on age, primary site, bone/marrow involvement, and number of metastatic sites [11], our patient accumulated two adverse factors, an unfavorable primary site and bone involvement. Using the more recent pooled European risk stratification that regroups patients as having ≤2 versus ≥3 Oberlin risk factors, this placed him in the more favorable subgroup (46.1% versus 12.5% 3-year event-free survival) [12]. His death about one year after diagnosis, despite this comparatively favorable stratification, illustrates the limits of population-based scores for an individual patient, and confirms the reputation of the retroperitoneal site as one of the most unfavorable for RMS [5,6], related less to poor chemosensitivity, given the striking initial regression, than to anatomical constraints precluding both wide resection and adjuvant irradiation, which could not be delivered here given the extensive disease at diagnosis.

Beyond diagnosis, imaging also guided treatment monitoring. Using the 3D-EpSSG volumetric method described by Orsatti et al. [13], based on three orthogonal tumor measurements and an ellipsoid volume approximation, the estimated tumor volume decreased by 96.2% after induction chemotherapy, indicating a marked treatment response. Spinal MRI, which was not performed in our patient, could have better assessed intraspinal extension given the vertebral involvement [4]. Diffusion-weighted MRI may also have provided an early functional biomarker of chemotherapy response [14] and helped distinguish viable residual tumor from post-therapeutic changes [15]. This distinction was particularly relevant in our patient, as the residual tissue remained radiologically stable and was considered non-viable by the surgical team but was not biopsied intraoperatively. Fusion-status molecular testing was not performed. The marked treatment response supported the conservative surgical strategy adopted in our patient, consistent with current function-preserving resection trends [16]. For local control in similarly extensive presentations, proton therapy, with its favorable dose distribution and greater sparing of surrounding normal tissues compared with conventional photon techniques, could also be considered where available [17]. This illustrates the gap that can exist between recommended multimodal work-up and real-world resource constraints.

This report is limited by its single-case, retrospective design and by the lack of MRI evaluation and molecular testing. In addition, no imaging was available immediately before death to determine whether the terminal clinical deterioration was associated with disease progression. It nonetheless underscores the value of close multidisciplinary collaboration between pediatric oncologists, radiologists, surgeons, and pathologists in the management of this rare tumor.

## Conclusions

Retroperitoneal RMS in children is rare but carries one of the poorest prognoses among primary RMS sites, related less to intrinsic chemoresistance than to anatomical constraints that limit complete locoregional control. This case illustrates the diagnostic difficulty of large retroperitoneal masses, the limitations of percutaneous biopsy in heterogeneous tumors, and the potential added value of MRI for assessing intraspinal extension and characterizing residual post-treatment tissue. It also emphasizes the importance of integrating imaging findings with pathological results, particularly when biopsy findings are discordant. Awareness of these imaging challenges and close multidisciplinary collaboration remain essential to optimize care for this rare and aggressive tumor.
